# Boosting Immunity against Multiple Myeloma

**DOI:** 10.3390/cancers13061221

**Published:** 2021-03-11

**Authors:** Raquel Lopes, Bruna Velosa Ferreira, Joana Caetano, Filipa Barahona, Emilie Arnault Carneiro, Cristina João

**Affiliations:** 1 Lymphoma and Myeloma Research Programme, Champalimaud Centre for the Unknown, 1400-038 Lisbon, Portugal; raquel.lopes@research.fchampalimaud.org (R.L.); bruna.ferreira@research.fchampalimaud.org (B.V.F.); joana.caetano@research.fchampalimaud.org (J.C.); filipa.barahona@research.fchampalimaud.org (F.B.); emilie.carneiro@research.fchampalimaud.org (E.A.C.); 2 Faculty of Medicine, University of Lisbon, 1649-028 Lisbon, Portugal; 3 Faculty of Medical Sciences, NOVA Medical School, 1169-056 Lisbon, Portugal; 4 Hemato-Oncology Department, Champalimaud Centre for the Unknown, 1400-038 Lisbon, Portugal

**Keywords:** multiple myeloma, immunotherapy, bone marrow immune microenvironment

## Abstract

**Simple Summary:**

Multiple myeloma is a hematological malignancy arising from the proliferation of tumor antibody-producing cells in the bone marrow. In this cancer, tumor growth is facilitated by a permissive bone marrow microenvironment including dysfunctional immune cells that lose their ability to control cancer cells and become “toxic friends”. Immunotherapy is a form of cancer treatment that aims to stimulate the immune system to “fight back” tumor cells. Several immunotherapies have been approved for clinical use and many others are currently under clinical trials. In this review, we focus on current and future immunotherapies used in multiple myeloma with impact on bone marrow immune microenvironment cells, also known as the bone marrow immunome.

**Abstract:**

Despite the improvement of patient’s outcome obtained by the current use of immunomodulatory drugs, proteasome inhibitors or anti-CD38 monoclonal antibodies, multiple myeloma (MM) remains an incurable disease. More recently, the testing in clinical trials of novel drugs such as anti-BCMA CAR-T cells, antibody–drug conjugates or bispecific antibodies broadened the possibility of improving patients’ survival. However, thus far, these treatment strategies have not been able to steadily eliminate all malignant cells, and the aim has been to induce a long-term complete response with minimal residual disease (MRD)-negative status. In this sense, approaches that target not only myeloma cells but also the surrounding microenvironment are promising strategies to achieve a sustained MRD negativity with prolonged survival. This review provides an overview of current and future strategies used for immunomodulation of MM focusing on the impact on bone marrow (BM) immunome.

## 1. Introduction

Multiple myeloma (MM) is an incurable hematological malignancy of mature B lymphocytes characterized by the accumulation of clonal plasma cells (PCs) within the bone marrow (BM). One of the hallmarks of MM is the progressive immune dysfunction and can be further associated with impaired anti-tumor immune responses, induction of angiogenesis and drug resistance [1,2,3], as described in Table 1.

**Figure 1:**
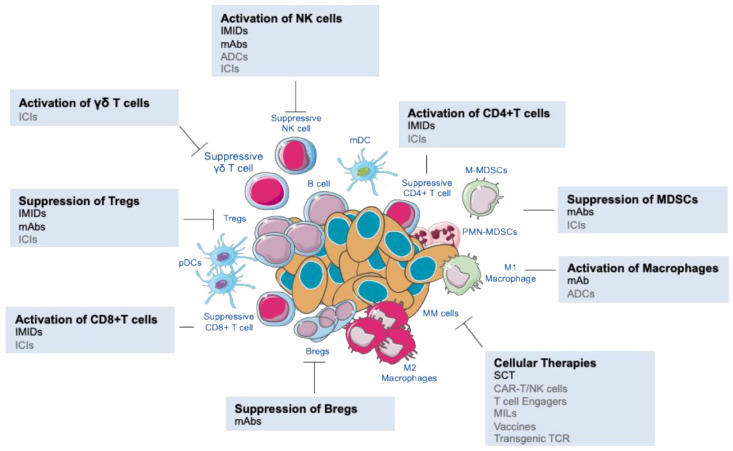
Overview of different immunotherapeutic approaches targeting the suppressive MM BM immune microenvironment and boosting innate and/or adaptive anti-tumor responses. NK, natural killer; M-MDSCs, monocytic MDSCs; PMN-MDSCs, polymorphonuclear myeloid-derived suppressor cells; Bregs, regulatory B cells; Tregs, regulatory T cells; pDCs, plasmacytoid dendritic cells; mDCs, myeloid DCs); IMIDs, Immunomodulators; mAbs, monoclonal antibodies; ADCs, antibody–drug conjugates; ICIs, immune checkpoint inhibitors; SCT, stem cell transplantation; CAR, chimeric antigen receptor; MILs, marrow-infiltrating lymphocytes; TCR, T cell receptor. Activation and suppression of immune populations showed by a line and stop line, respectively. Immunotherapies that are under development for MM are listed in grey.

However, our increased knowledge of the pathophysiology of MM has shown that immunotherapy holds great promise to treat this hematological disease. As such, several immunotherapies targeting immune cells have been approved for clinical use and many others are currently under clinical trials. In this review, we focus on current and future strategies used for immunomodulation of MM focusing on the impact on BM immunome, including approved strategies, breakthrough therapies and future approaches (Figure 1).

## 2. Approved Immune Strategies in MM

### 2.1. Stem Cell Transplantation

Despite the emergency of novel agents in the last years, autologous stem cell transplantation (ASCT) remains the standard of care for young patients with newly diagnosed MM [16]. Immunological changes associated with ASCT suggest that disease plateau after transplant may arise from more than just tumor reduction after high-dose chemotherapy. The use of high doses of melphalan has been shown to induce immunogenic cell death, increase of inflammatory cytokines production, enhance tumor antigen uptake by dendritic cells (DCs) with subsequent antigen presentation to lymphocytes [17]. In addition, it is known that the reconstitution after ASCT alters CD4+/CD8+ T cell ratio. This can either provide a favorable effector T cell/Treg ratio [18] or reconstitute exhausted CD8+ T cell populations, as reported by Minnie et al. [19]. This may support the use of immune checkpoint inhibitors (ICIs) after ASCT in MM.

On the other hand, allogeneic stem cell transplantation (AlloSCT) may lead to long-term progression-free survival (PFS) and might be considered as a potential curative option for myeloma patients [20]. However, its use remains limited due to high risk of treatment-related mortality (TRM), low graft vs. MM (GVM) effect, graft-versus-host disease (GvHD) and/or immunosuppression followed by infections [21,22]. Several trials have already demonstrated that this therapeutic approach can lead to good clinical responses in high-risk myeloma patients [23,24,25]. Nonmyeloablative reduced-intensity conditioning (RIC) aims at minimizing TRM and toxicity, relying more on exploring GVM for anti-myeloma efficacy. A recent manuscript published by Gahrton et al. critically reviews the good results of RIC AlloSCT and how it could be combined with proteasome inhibitors, immunomodulators, monoclonal antibodies (mAbs) and cellular immune therapies [26]. The strategy of conducting RIC AlloSCT after ASCT (Auto/RICAlloSCT) aiming at debulking with the initial autologous and using the tandem RICAllo to generate GVM effect has been followed by several groups with good results. Recently, a pooled analysis was performed on individual patient data from the Italian-Torino (*n* = 162), Spanish PETHEMA (*n* = 110), EBMT-NMAM2000 (*n* = 357) and BMT-CTN (*n* = 709) studies [27]. There were 1338 patients included, 439 in the Auto/RICAllo group and 899 in the Auto/Auto group. In this updated analysis, the median follow-up of survivors was 118.5 months. Overall survival (OS) was 62.3% vs. 59.8% at five years and 44.1% vs. 36.4% at ten years (*p* = 0.01) for Auto/RICAllo and Auto/Auto, respectively. Because the availability of matched unrelated donors is not so big, especially for non-Caucasian patients, the use of haploidentical donors has been increasing to perform AlloSCT in MM. A retrospective study of 96 patients receiving haploidentical transplants as salvage treatment between 2008 and 2016, reported to the European Society for Blood and Marrow Transplantation (EMBT) and the Center for International Blood and Marrow Transplant Research (CIBMTR) registries, showed interesting results. All patients had received 1–3 previous ASCT. TRM at one year was 21% and PFS and OS at two years were 17% and 48%, respectively [28]. Although the follow-up of this study is short, these results show that haploidentical AlloSCT is feasible as salvage therapy in MM, achieving quite similar results to those with matched unrelated donors.

Beside BM stem cells grafts, cord blood may also be a feasible alternative graft source for MM patients. A registry database study of myeloma patient who underwent cord blood transplantation was recently published and showed a cumulative incidence of TRM at two years higher than what is reported in latest studies on RICAlloSCT for MM [29].

Despite these results, AlloSCT still lacks a consistent benefit and some specialists have already insinuated that, with all the anti-myeloma therapies currently available, the time of AlloSCT in MM is over [21]. Indeed, this treatment strategy is not considered as standard of care for newly diagnosed or relapsed standard risk MM patients.

Overall, we believe that, in an era where myeloma treatment is drastically changing with the development and approval of novel immunotherapeutic agents, the combination of SCT with these therapies may be the focus of future transplant clinical trials in MM.

### 2.2. IMIDs

Immunomodulators (IMIDs) (e.g., thalidomide, lenalidomide and pomalidomide) have contributed to a significant improvement of MM patient outcomes and are currently the backbone of several MM treatment regimens. These agents have both tumor- and immune-targeting effects [30]. IMIDs act through cereblon-dependent degradation of both Ikaros (IKZF1) and Aiolos (IKZF3) resulting in direct myeloma cell apoptosis [31,32]. Their impact on BM immune cells is a favorable off-target effect by stimulating T and NK cells’ activity. Particularly, IMIDs increase proliferation, enhance Th1 cytokine production (IL-2 and IFN-γ) and reduce IL-10 secretion by CD4+ and CD8+ T cells, resulting in NK cell activation, expansion and antibody-dependent cellular cytotoxicity (ADCC) [30,33]. In vitro studies showed that IMIDs also suppress Treg expansion [34]. Other immune-modulating properties of IMIDs include the potentiation of mAb therapy. Recently, it was described that these agents prime myeloma cells for killing with daratumumab (mAbs section) and sensitize myeloma cells to NK cell-mediated ADCC [35]. In the concept of improving responses using combinatory strategies, lenalidomide, which is a second generation IMID and an analog of thalidomide, is also being explored in combination with vaccination approaches in clinical trials, increasing immunogenicity [36,37,38]. On the other hand, a study from Gandhi et al. demonstrated that, although dexamethasone inhibits myeloma cell growth, it also reduces lenalidomide-mediated lymphocytes (T and NK) activation [39].

More recently, a novel class of immunomodulatory drugs called CEreblon E3 Ligase MoDulators (CELMoDs) is being developed. These agents work through a fast and more effective degradation of Ikaros and Aiolos. Iberdomide (CC-220) is one of the next generation cereblon target agents that have shown strong immunomodulatory activity. In vitro, CC-220 has enhanced anti-proliferative and tumoricidal activity against MM cell lines, including those resistant to lenalidomide and pomalidomide, with strong immune stimulatory activity [40]. Preliminary results from a phase 1b/2a of iberdomide in combination with dexamethasone presented at the American Society of Clinical Oncology (ASCO) in 2019 demonstrated efficacy and safety of this combination in heavily pretreated relapsed/refractory (RR) MM patients [41]. This study is ongoing, and many other synergies are being tested, namely iberdomide plus the mAb daratumumab or in combination with bortezomib (NCT02773030). 

Furthermore, CC-92480, another CELMoD agent, has shown outstanding preclinical results with great anti-myeloma and immune-stimulatory activity [42]. These results supported its translation into a first-in-human study of this CELMoD agent plus dexamethasone in RR MM patients (NCT03374085), and early-phase findings show encouraging efficacy [43].

We consider that future clinical trials will continue to explore potential combinations between IMIDs and other novel immunotherapeutic agents or even with conventional chemotherapies.

### 2.3. PIs

Proteasome inhibitors (PIs), widely used and one of the backbones in MM therapy, are designed to disrupt the natural degradation of intracellular proteins via the proteasome, inducing direct apoptosis of neoplastic plasma cells. However, apart from their cytotoxicity activity, PIs exert their biological activities through the inhibition of cytokines’ secretion, suppression of several adhesion molecules and prevention of angiogenesis [44]. Presently, three classes of agents have been approved—bortezomib, carfilzomib and ixazomib. 

Bortezomib was the first PI approved for the treatment of newly diagnosed and RR MM patients. This agent works through several mechanisms including the inhibition of NFkB pathway, thereby blocking the activation of anti-apoptotic genes; upregulation of NOXA, resulting in myeloma cell death; or the stimulation of osteoblasts that are involved in bone formation [45]. Furthermore, several preclinical experiments already demonstrated that bortezomib induces immunogenic cell death (ICD) by several mechanisms, namely through the downregulation of the expression of HLA class I that stimulates NK-mediated myeloma cell lysis [46]. More recently, Gullà et al. made an extensive study on the mechanisms and clinical outcomes induced by bortezomib-mediated ICD. Specifically, they found that in vitro exposure to bortezomib therapy resulted in an activation of DCs through calreticulin and engulfment of myeloma cells. The importance of the immune system was further validated in vivo and through single cell analysis using samples from myeloma patients [47]. Overall, these results suggest that the use of bortezomib, even as single therapeutic agent, is able to boost an immunological response against MM. Of note, considering its powerful effect, this drug has been widely used in the treatment of every stage of myeloma disease [48].

Carfilzomib is a second-generation PI currently used in the treatment of RR MM patients and exerts its activity through inhibition of cell proliferation and stimulation of tumor cell death. Both Food and Drug Administration (FDA) and European Medical Agency (EMA) approved this second-generation PI for the treatment of RR MM patients in combination and only FDA approved it as monotherapy [49]. The CANDOR study (NCT03158688) is an active phase III clinical trial currently evaluating the potential of a triple combination using dexamethasone, daratumumab and carfilzomib (experimental) vs. dexamethasone plus carfilzomib (control) in RR MM patients, with already good published results [50]. This demonstrates a favorable risk–benefit profile and suggests that this triplet combination could be introduced into the armamentarium of treatments for RR MM patients. Other combination regimens using carfilzomib are currently being investigated, namely carfilzomib plus cyclophosphamide and dexamethasone (NCT03336073) or carfilzomib and hydroxychloroquine (NCT04163107) in RR MM patients.

Ixazomib is another second-generation PI, and it was the first oral PI to be approved for RR MM patients. It works mainly through induction of apoptosis by the activation of both caspase-8 and -9, cell cycle arrest, blocking NFkB signaling pathway and inhibiting angiogenesis [51]. In a phase III clinical trial, the addition of this PI to lenalidomide and dexamethasone in the treatment regimen of RR MM patients showed longer PFS compared to those that did not received ixazomib [52]. Results are also very promising in naive MM patients when combined with lenalidomide and low-dose dexamethasone [53]. Other combinatory strategies are being evaluated in clinical trials, including maintenance therapy with oral ixazomib in newly diagnosed patients who were not treated with SCT (NCT03748953), ixazomib plus daratumumab without dexamethasone in elderly RR MM patients (IDARA study—NCT03757221) and the combination of ixazomib, lenalidomide and dexamethasone in smoldering MM (SMM) (NCT02916771).

Ultimately, there is no doubt that PIs are crucial to increase survival and maintain quality of life in MM. Indeed, patients are achieving deeper responses, remarkably those with poor prognosis. In addition, other second-generation PIs are being developed and tested, including marizomib (NPI-0052), oprozomib (ONX0912) and delanzomib (CEP-18770) [54].

### 2.4. Monoclonal Antibodies

The introduction of antibody-based therapies has been considered a great improvement in MM treatment due to its clinical results, even in patients with advanced disease [55]. In general, mAbs act through a variety of mechanisms, establishing a link between tumor cells and immune effector cells, leading to myeloma cell death through ADCC, antibody-dependent cellular phagocytosis (ADCP) and via complement-dependent cytotoxicity (CDC) by which mAbs activate a cascade of proteolytic enzymes that ultimately rupture tumor cell membrane [56]. Recently, FDA approved antibodies targeting both CD38 (daratumumab and isatuximab) and SLAM7 (elotuzumab) for MM treatment [57].

Daratumumab has pleiotropic mechanisms of action, including direct apoptotic activity, macrophage-mediated phagocytosis, Fc-dependent immune-effector and immunomodulatory effects. By eradicating CD38-expressing MDSCs, Tregs and Bregs, daratumumab potentially restores anti-tumor immune responses with an increase in NK and T cells expansion, activation and clonality [58,59,60,61]. Furthermore, daratumumab has an ectoenzymatic activity that is involved in the generation of adenosine, known to disrupt immune homeostasis in MM context and this suppressive effect has been described on DCs and lymphocytes, namely T and NK cells [62].

Several studies have clearly demonstrated the ability of daratumumab induce a potent cytotoxic activity both in vitro and in vivo [59], which gave all the rationale to translate these findings to the first-in-human tests. As such, results from the GEN501 and SIRIUS clinical trials led to daratumumab’s approval as a single agent for RR MM patients after demonstrating rapid, deep and durable responses [63,64,65]. Of note, daratumumab has shown outstanding effects in RR MM patients, especially in combination with other backbone agents in MM therapy, including lenalidomide/dexamethasone (Rd) or bortezomib/dexamethasone [66,67].

Additionally, Nihjof and colleagues published that CD38 expression levels in pretreated MM patients could work as a biomarker of response against the anti-CD38 mAb daratumumab. Furthermore, when patients become resistant to this therapeutic approach, researchers found that there was an increase in the expression levels of both complement-inhibitory proteins CD55 and CD59. However, this effect was reversed with all-trans retinoic acid (ATRA) and resulted in a significantly improved anti-CD38 mAb-mediated complement-dependent cytotoxicity [68]. This study suggests that a combination with daratumumab and ATRA should be explored in future clinical trials. Indeed, a clinical trial trying to develop a safe combination of daratumumab plus ATRA suitable for clinical use is currently active (NCT02751255). Importantly, other clinical trials are presently under evaluation using daratumumab in combination with other agents, such as anti-PD-1 mAb (NCT03357952) in RR MM patients or the addition of daratumumab to standard VRd therapy (bortezomib, lenalidomide and dexamethasone) in naive MM patients and for whom SCT is not planned (NCT03652064).

Isatuximab is another anti-CD38 mAb, which has similar binding affinity to myeloma cells and activity. It induces MM apoptosis via the classic caspase-dependent apoptotic pathway and lysosomal cell death pathway [69]. This mAb was recently approved for the treatment of RR MM patients in combination with pomalidomide and dexamethasone [70]. Recently, preliminary results from a phase II clinical trial (NCT01084252) have demonstrated superior anti-tumor activity of the combination of dexamethasone and isatuximab compared to this anti-CD38 mAb alone in MM patients with at least three prior lines of therapy. Importantly, there was no significant decrease on safety [71,72]. Isatuximab has also shown promising results when combined with IMIDs in the first line of treatment or in RR MM patients [73,74]. An active clinical trial is currently evaluating the safety, pharmacokinetics and efficacy of this mAb in RR MM patients and results are eagerly awaited (NCT02514668).

Elotuzumab, which is an anti-SLAM7 mAb, promotes MM killing mainly through NK-mediated ADCC and can also prevent adhesion of myeloma cells to BM stromal cells [75]. Moreover, when elotuzumab binds to myeloma cells, the Fc portion of this mAb binds to its receptor on NK cells (CD16), triggering an even stronger NK cell cytotoxic activation [76]. However, given that this mAb only acts through NK cells, its clinical effect on RR MM patients is limited as a single agent, possibly due to NK impairment in these patients. Nonetheless, by adding lenalidomide or pomalidomide and dexamethasone, phase III trials showed improved clinical efficacy. Indeed, ELOQUENT study led to its approval by FDA [77,78]. Moreover, the addition of elotuzumab to bortezomib and dexamethasone therapeutic regimen in a phase II study (NCT01478048) has shown an improvement of PFS in RR MM patients compared to patients who received bortezomib and dexamethasone alone and a significant decrease in the risk of disease progression or death in patients treated with this triple regimen [79]. A phase III clinical trial is currently recruiting newly diagnosed MM to test the addition of the mAb Elotuzumab to the triple induction regiment KRd, which comprises carfilzomib, lenalidomide and dexamethasone, following ASCT and subsequent maintenance with elotuzumab plus lenalidomide, in order to see if patients can reach longer PFS (NCT03948035).

Taken together, we consider that the use of mAbs is an area in expansion with very encouraging results in MM setting. However, we are now noticing treatment-associated resistance [68,80,81] and alternative approaches need to be designed in order to overcome this issue, such as the use of a second antibody with the same specificity but recognizing a different epitope.

## 3. Breakthrough Immune Therapies in MM

### 3.1. CAR-T Cells

Chimeric antigen receptor T (CAR-T) is a genetically modified T cell that expresses a specific receptor-targeting antigen on its surface, independently of HLA presentation [82]. Autologous CAR-T cells have presented unprecedented results in patients with B cell hematological malignancies that were considered incurable and led to the approval of two anti-CD19 CAR-T cells: tisagenlecleucel and axicabtagene ciloleucel. These products have been used for the treatment of RR B cell acute lymphoblastic leukemias, and RR diffuse large B cell lymphomas and primary mediastinal large B cell lymphomas, respectively [83,84].

In MM, numerous targets are currently being tested in clinical trials and some of them already show encouraging results. These include CD19 and/or B-cell maturation antigen (BCMA) (NCT03767725). Of note, because BCMA is expressed in myeloma cells and, more importantly, it is largely restricted to plasma cells and some mature B cells, the first CAR-T to be developed for MM targeted this surface protein [85]. BCMA-targeted CAR-T cells have produced very promising results in phase I clinical trials in RR MM patients, with many reporting overall response rates (ORRs) of 64–88% in a difficult-to-treat patient cohort [86,87,88]. Many other trials are currently ongoing for RR MM patients testing either CAR-T cell therapy alone (NCT03090659 and NCT02658929) or in combination (NCT03318861).

Encouraging CAR-T cell targets in myeloma include, among others, CD138 (NCT03672318), SLAMF7 (NCT03958656) and GPRC5D (NCT04555551). These targets are all in early stages of testing, but preclinical and in vitro studies have shown promising activity against tumor cell lines and primary myeloma cells [89,90,91].

Despite impressive responses early after CAR-T cell infusion in phase I clinical trials, the lack of persistence and durability of these cells in MM patients, unlike lymphoma, are being further investigated. This can be due to intrinsic factors related to the CAR-T, such as binding affinity or manufacturing, or tumor intrinsic factors, such as loss of target antigen or even the immunosuppressive BM microenvironment [92,93]. In addition, early clinical formulations of CAR-T cells were generated without regard for phenotype or functional heterogeneity in leukapheresis products. However, a clinical study found that higher frequencies of CD8+ T cells with a naive or stem memory phenotype in the leukapheresis product correlated with a better outcome [88]. Importantly, the frequency of early memory T cells was reduced in T cell products from heavily pre-treated patients, suggesting that collection of T cells at earlier stages of disease might prove beneficial [94]. Another practical limitation of autologous CAR T-cell therapy is the manufacturing process time that may delay prompt treatment of myeloma aggressive disease. To overcome this, allogeneic CAR-T cell therapy is being pursued allowing the production of an “off-the-shelf” treatment. Recently, the FDA has granted fast track designation to PBCAR269A, Precision BioSciences’ donor-derived CAR-T cell therapy, for the treatment of RR MM. A phase I/IIa trial (NCT04171843) testing the use of allogeneic anti-BCMA CAR-T cell is currently recruiting MM patients refractory to two prior treatment regimens, including an IMID or PI.

### 3.2. CAR-NK Cells

Another type of CAR cell therapy that is giving its first steps in MM is CAR-NK. In this case, the approach is not limited by autologous manufacturing since NK cells are not HLA-restricted and can be produced by other means, such as NK cell lines (e.g., NK92), umbilical cord blood or induced pluripotent stem cells. CAR-equipped NK cells have some advantages over CAR-T cells, which include “off-the-shelf” products, no risk of GvHD and lower toxicity and cost [95,96]. Although most clinical trials in MM using this approach are still limited to pre-clinical studies, some trials are already ongoing. For instance, a trial in RR BCMA+ MM patients began in 2019 in which subjects are treated with anti-BCMA CAR-NK92 cells (NCT03940833). Until now, this is the only clinical trial using CARs targeting NK cells in MM, but it has already produced interesting and safe results in CD19+ B cell malignancies [97].

### 3.3. T Cell Engagers

Bi-specific T-cell engagers (BiTES) represent the pioneering subclass of T cell engager molecules currently being developed as novel immunotherapeutic option for MM patients. These drugs can simultaneously bind to two different epitopes on both T and tumor cells, resulting in T cell activation and simultaneous myeloma cell lysis [98]. Presently, no BiTE has been approved for MM patients. Considering the highly encouraging pre-clinical results, there are more than a dozen clinical trials evaluating the effects of these bi-specific agents against MM-specific antigens, such as BCMA, CD38, CD19, GPRC5D and FcRH5 [99]. The most clinically advanced developed anti-BCMA BiTE to treat MM is the AMG 701. This BiTE has an extended half-life and has showed strong encouraging results [100]. A trial is currently recruiting RR MM patients to receive this therapy either as monotherapy or in combination with IMIDs (e.g., pomalidomide) and with or without dexamethasone (NCT03287908) [101]. BI 836909 is another T cell engager that has demonstrated successful in vivo anti-myeloma activity [102]. This compound was recently tested as monotherapy in last line RR MM patients, but results have not been published yet (NCT02514239).

It is important to note that BiTES rely on the presence of a functional T cell response, and this therapy is likely to be most efficacious after ASCT or in newly diagnosed MM patients. Nonetheless, an early-phase trial demonstrated promising efficacy of a BCMA-CD3 bispecific antibody—CC-93269—in heavily pretreated patients with an ORR of almost 90% after treatment at the highest dose bracket [103].

Although this therapeutic approach has shown promising results in both pre-clinical and early clinical trials, unfortunately there are several barriers until its approval, mainly due to inherent treatment-related toxicity (e.g., cytokine release syndrome) or hallmarks of tumor evasion (downregulation of antigens) [104,105]. Thus, it is imperative to optimize this technique for a safer and more effective application.

Lately, other formulations have been developed in order to improve engagers’ efficacy, namely BiKES that redirect NK cells against myeloma cells through CD16A and tumor-specific antigens, tumor-targeted immunomodulators that target both co-stimulatory molecules and tumor antigens, and dual immunomodulators that target either inhibitory and/or activation markers simultaneously [106]. More recently, a novel class of engagers has emerged: trispecific antibodies (TriTES or TriKES, depending on targeted lymphocyte). Even though there are no clinical trials currently ongoing in MM setting, preclinical trials have demonstrated increased efficacy over BiTES [107].

Particularly, a novel bispecific antibody is currently being evaluated. CC-93269 (EngMab) is a humanized 2 + 1 IgG1-based T cell engager that binds to BCMA in myeloma cells and to CD3ε on T cells and has been shown to induce tumor regression in MM preclinical models [108]. Considering these results, a phase I clinical trial is currently ongoing in RR MM patients (NCT03486067), with exciting first results presented at the American Society of Hematology (ASH) in 2019 [103].

In brief, the field of bispecific antibodies is growing and holds great promise as a novel immunotherapeutic approach for MM, paving the way towards a better control of the disease.

### 3.4. ADCs

A novel type of immunotherapy that is also being developed and tested in MM are antibody drug conjugates (ADCs). These conjugates consist of a mAb that carries a cytotoxic drug, also known as payload (e.g., chemotherapy), and, when it reaches its programmed target on myeloma’s surface, it releases the chemotherapy agent, resulting in cell death [109]. Furthermore, it was also reported that some immunoconjugates kill myeloma cells through ADCC or ADCP [110,111]. Importantly, this mechanism of action limits damage of healthy cells and subsequently reduces chemotherapy-associated side effects [109]. However, in some cases, a bystander killing effect of non-tumor cells, including immune populations, might happen, even if they do not express the target antigen. This effect can occur before the drug is internalized through the release of extracellular enzymes (e.g., cathepsin B) by tumor cells or TAMs. These enzymes will cleavage and generate a diffusible drug from the ADC, afterwards uptaken by surrounding healthy cells and resulting in cell death. Other mechanisms of bystander killing of immune populations are through the secretion of the free drug to the extracellular space after internalization and degradation of the ADC by the tumor cells [112].

Presently, there are several ADCs under clinical development for MM, particularly for RR patients. These targets include anti-CD56 (lorvotuzumab mertansine; NCT00346255) [113] or anti-CD138 (indatuximab ravtansine; NCT01001442). These agents were also tested in combination with other therapies, such as dexamethasone and/or lenalidomide, and were found to be well tolerated (NCT00991562 and NCT01638936, respectively) [114,115]. Anti-CD38, -CD46 and -CD74 are other examples of targets that are currently being explored in MM.

Anti-CD38 ADCs have shown promising anti-myeloma activity. Phase I clinical trials are starting to evaluate safety and efficacy of both TAK-169 (NCT04017130) and -573 (NCT03215030) in RR MM patients. While TAK-169 consists of a ribosome inactivating Shiga-like toxin A-subunit (SLTA), TAK-573 incorporates an attenuated form of IFN-α [116,117].

CD46 is known to be highly expressed in MM cell lines and it has been shown that fusing with saporin, and consequently to monomethylauristatin F (MMAF), resulted in myeloma cell killing both in vitro and in vivo [118]. The anti-CD46 ADC FOR46 is conjugated with a yet undisclosed cytotoxic payload and is being tested in a phase I clinical trial for RR MM patients (NCT03650491).

CD74 is also an attractive therapeutic target because it is expressed in more than 90% of B cell malignancies and is rapidly internalized and recycled [119]. Milatuzumab doxorubicin (hLL1-DOX) has been tested in patients with RR disease, but phase I/II clinical trial was interrupted due to lack of efficacy (NCT01101594). Another anti-CD74 ADC with anti-tumor activity in preclinical studies is STRO-001, currently under recruitment in clinical trials for advanced B cell malignancies, including for RR MM patients (NCT03424603) [120].

Considering that BCMA expression is mainly restricted to a subgroup of mature B cells and neoplastic plasma cells, this molecule constitutes an attractive target for MM therapy [121]. Hence, an anti-BCMA ADC—belantamab mafodotin (GSK2857916)—has demonstrated meaningful single-agent activity in phase II of DREAMM-2 study for heavily pre-treated patients with RR MM, leading to its approval as monotherapy by the FDA and EMA [122,123]. Given these results, more phase II/III clinical trials are presently ongoing to test this agent together with other already approved therapies for RR MM (NCT03544281, NCT04246047 and NCT04484623). MEDI2228 is another anti-BCMA ADC fused with pyrrolobenzodiazepine via a protease-cleavable linker. Pre-clinical tests, either as monotherapy or in combination with other agents (e.g., bortezomib), have shown anti-myeloma activity further supporting its translation into clinical trials for RR MM patients (NCT03489525) [124,125,126]. Many other BCMA-targeting ADCs are currently being evaluated in RR MM patients, including AMG-224 (NCT02561962) or CC-99712 (NCT04036461).

Another potential target of interest in neoplastic plasma cells is ICAM-1. Although the naked mAb anti-ICAM-1 showed limited efficacy, researchers believe that, by conjugating this antibody, its anti-myeloma activity can be improved. In a very recent preclinical work, Sherbenou and colleagues showed that anti-ICAM fused with an autistatin derivative displayed potent cytotoxic activity. Given these results, the team proposes to begin clinical trials in RR MM patients [127].

In summary, although these chemo-immunotherapies seem to be an important modality for cancer treatment, clinical trials need to be grounded in better and more extensive preclinical data and carefully designed since some of them had to stop due to either low efficacy or high levels of toxicity (e.g., bystander effect).

## 4. Future Approaches in MM Treatment

### 4.1. Immune Checkpoint Inhibitors

As aforementioned, myeloma cells can switch off T cells’ anti-tumor responses through a wide spectrum of mechanisms. Thus, several agents targeting these immune checkpoints have been recently developed as a strategy to overcome immune evasion in MM context [128].

However, activating the immune system has risks. Several studies suggested the importance of the axis PD-1/PD-L1 in MM giving strength to the use of ICIs [129,130,131], but results from clinical trials have been discouraging. Unlike in solid tumors, PD-1 blockade in RR MM patients has no activity as a single agent, with early clinical studies reporting a lack of efficacy of nivolumab as monotherapy [132]. This suggested that IMIDs, such as lenalidomide or pomalidomide, could be necessary in combination with anti-PD-1 blockade to increase depth and duration of response post-ASCT. To explore this, a phase II clinical trial (NCT02906332) using pembrolizumab early after ASCT followed by lenalidomide recently reported an improvement in the CR rate in high-risk myeloma patients [133]. However, the phase III studies (NCT02289222 and NCT02036502) of anti–PD-1/PD-L1 agents with IMiDs in patients with RR MM have resulted in substantial toxicity and no improvement in objective response rates, such that several trials were placed on a clinical hold by the FDA and subsequently ended. A phase Ib/IIA trial investigating ipilimumab in combination with nivolumab early after ASCT in MM patients with high risk of relapse reported a promising PFS rate (NCT02681302). However, with this combination of ICIs, most patients developed immune-related adverse events grade 2 or higher and required treatment with systemic steroids [134]. A phase I clinical trial combining anti–IL-17A with PDR001 (anti–PD-1) in RR MM patients (NCT03111992) is another hypothesis already tested and results are further warranted.

Another ICI that is known to negatively regulate lymphocytes’ function is TIGIT [135]. Presently, there are only two clinical trials recruiting to test anti-TIGIT potential in MM either alone (NCT04354246) or in combination for patients with RR MM (NCT04150965).

Overall, although preclinical studies provide evidence that targeting ICIs might be a conceivable strategy to improve MM therapy, a major scientific and clinical challenge remains unanswered: finding an equilibrium between myeloma cells killing efficacy and toxicity.

### 4.2. MILs

Another cellular-based therapy emerging in MM is the use of marrow-infiltrating lymphocytes (MILs) as a source of T cells for adoptive cell therapy. MILs have been shown to be a particularly rich source of myeloma-specific cytotoxic and memory T cells owing to exposure to malignant plasma cells in BM [136]. Definitely, the distinctive biology of the BM microenvironment is able to shape MILs, providing a rationale to use these cells therapeutically. Furthermore, considering this unique milieu, T cells coming from BM might be a better source compared to lymphocytes collected from peripheral blood (PB). Hence, these infiltrating lymphocytes may play a key role in the development of more effective and safe immunotherapies for MM.

Indeed, using NOD/SCID mice, Borrello et al. demonstrated that, upon infusion, MILs traffic to the BM where they persist and exert potent anti-tumor activity [137]. In accordance with this preclinical finding, clinical data show a direct correlation between complete response (CR) early after ASCT and the presence of tumor-specific immunity against myeloma cells. Even though there was persistence of MILs in the BM one year after adoptive cell transfer (ACT), the durability of clinical response was still limited. Notwithstanding, this study proved the efficacy and feasibility of ACT using MILs [138]. In another preclinical study, Lutz and colleagues demonstrated that the use of MILS in CAR technology (CAR-MILs) improved in vitro myeloma cell killing compared to PB lymphocytes (PBLs). Particularly, they found that this therapeutic approach enhanced cytotoxic function, increased stemness and reduced immune exhaustion. These preliminary data suggest that CAR-MILs might provide a more robust anti-tumor response compared to general CAR-PBLs [139]. Currently, there is one active phase II clinical trial assessing the efficacy of MILs alone or in combination with an allogeneic myeloma vaccine (NCT01045460) in MM patients that have finished induction therapy and are eligible for ASCT.

Altogether, we can conclude that MILs represent a hopeful tumor specific ASCT strategy not only for MM but also for other hematological neoplasms.

### 4.3. Vaccine Strategies

Another strategy to enhance anti-cancer immunity could be the use of vaccines [140]. DC-based immunotherapy holds great promise in several types of cancer, including MM [141]. Indeed, several vaccination approaches have been tested in this neoplasia, including idiotype-based, DC-based, myeloma cell lysates, myeloma-dying cells, DC-myeloma hybrids, cancer testis antigen–based (e.g., melanoma-associated antigen 3 (MAGE-A3) or New York esophageal squamous cell carcinoma-1 (NY-ESO-1)) and granulocyte-macrophage colony-stimulating factor (GM-CSF) cellular-based vaccines [142]. Unfortunately, although there is pre-clinical evidence of success, clinical translation has been proven to be challenging, since preliminary results lack efficacy [143,144]. This difficulty has been mainly attributed to the fact that DCs have impaired immune functions within the tumor bed [145,146,147]. Thus, in an attempt to increase DC-based power, it is imperative to develop novel ways to overcome antigen immunogenicity and tumor suppression as means of providing a more vigorous and maintained immune response with clinical effect. This would include the search for alternative sources of DCs or their combination with other agents that target the BM microenvironment (e.g., Tregs depletion or anti-ICIs). As such, the combination of immunomodulatory agents with anti-myeloma vaccination has been explored and has shown better results. For example, Luptakova et al. found that lenalidomide augments T cell responses to the DC/myeloma cells’ fusion vaccine through the production of inflammatory cytokines (e.g., IFN-γ) and cytotoxic lysis of autologous MM targets [33]. Additionally, a study incorporating idiotype-pulsed DC vaccination (Mylovenge—APC8020) after ASCT demonstrated improved survival in treated patients compared with historical controls who underwent ASCT without vaccination at the same center during the same time period (median OS 5.3 vs. 3.4 years) [148].

Rosenblatt and colleagues developed a tumor vaccine with patient-derived tumor cells fused with autologous DCs and co-administered with GM-CSF to MM patients. In this phase I study, the researchers found that this vaccine cocktail was able to produce an anti-tumor response with the expansion of both CD4+ and CD8+T cells in 11 out of 15 patients. Importantly, autologous DC/myeloma fusion cells have shown to be well tolerated without generation of autoimmunity and no evidence of dose-limiting toxicity with disease stabilization in most MM patients with advanced disease (NCT00459069). More recently, DC/tumor fusion vaccine was tested in a phase II clinical trial in which patients underwent vaccination followed by ASCT to achieve minimal residual disease (MRD). This resulted in an expansion of both CD4+ and CD8+ tumor-specific T cells upon post-transplant vaccination. Seventy-eight percent of the patients achieved CR or very good partial response and 24% of those who had achieved a partial response converted to CR/near CR 100 days after transplant and vaccination. This late response suggests that DCs/tumor fusion vaccination in the early post-transplant stage has an impact in residual disease [149].

Interestingly, an early phase I clinical trial is also being prepared for patients with monoclonal gammopathy of undetermined significance (MGUS) or SMM (NCT03591614). In this study, researchers aim to determine the safety and efficacy of a DC DKK vaccine in patients with non-active disease to be further used as a novel strategy to prevent progression of asymptomatic plasma cell disorders, stabilize disease and eventually eradicate MM.

Globally, despite their limitations and although further trials are needed, preliminary data suggest that vaccination in combination with other therapeutic agents might be a conceivable strategy able to induce specific T cell responses and increase their effectiveness in cancer patients.

### 4.4. Transgenic TCR

Autologous transgenic TCR T cells are ex vivo-modified T cells to express TCRs targeting extracellular or intracellular tumor-associated antigens (TAAs), although with MHC-restricted recognition [150,151]. Of note, one of the hallmarks of escape immune recognition is the fact that tumor cells often downregulate MHC expression; thus, this can be a weakness of this approach [152]. Furthermore, off-target effects might also happen due to heterologous pairing with endogenous TCRs [153].

TCR-transduced T cell therapies are currently being investigated in several neoplasias, including MM. In particular, current research is targeting cancer testis antigens, namely NY-ESO1 or the intracellular B cell-specific transcription factor (BOB1).

A phase I/II clinical trial of transgenic TCR T cells targeting NY-ESO-1-LAGE-1 showed expansion and persistence of these genetically engineered T cells. In addition, anti-NY-ESO-1 TCR-engineered cells were well tolerated and exhibited anti-tumor activity. CR/near CR was seen in 14 out of 20 patients (70%), with a PFS of 19.1 months. Unfortunately, disease progression in 8 out of 10 patients was due to loss of T cell persistence [154]. More recently, Stadtmauer et al. reported the results from a clinical trial in which RR or high-risk MM patients received an infusion of NY-ESO-1 specific peptide enhanced affinity receptor (SPEAR) T cells. In this trial, the objective response rate at day 42 was 80% and 44% after one year, with median PFS of 13.5 months and OS of 35.1 months. Importantly, infusions were tolerated and NY-ESO-1 SPEAR T cells showed effective anti-tumor response within the BM [155]. TCR targeting BOB1 might be an important addition to current immunotherapeutic strategies, since it is expressed in several still incurable hematological neoplasias, such as acute lymphoblastic leukemia, chronic lymphocytic leukemia, mantle cell lymphoma and MM. Indeed, Jahn and colleagues showed that BOB1-specific TCRs were able to lyse MM cells in vivo and there was absence of reactivity towards BOB1 negative cells, suggesting no off-target toxicity [156]. As such, administration of BOB1-specific genetically engineered T cells could be a novel and attractive strategy for patients with B cell malignancies. However, no clinical trials are currently ongoing using this antigen.

Until now, despite clinical trials using TCR transgenic T cells showed encouraging results with sustained antigen-specific antitumor effects in MM, this immunotherapeutic strategy is still under clinical evaluation and remains unavailable.

The MM BM immune components targeted by the different immunotherapeutic options discussed in this review are summarized in Table 2.

## 5. Conclusions and Future Perspectives

As the mechanisms by which immune cells interact with tumor cells and protect them against standard anti-myeloma drugs are being deciphered, the list of future possible immunotherapeutic agents that are reaching promising results has significantly increased in the last years. However, this disease still lacks an effective treatment and remains incurable. Therefore, much work is still needed to translate new drugs from the bench to clinical trials and finally into the bedside. Notwithstanding, we believe that the solution might come from using combinatorial therapies. As we are witnessing in recent years, combo protocols show good results in terms of efficacy and survival benefit. Combinatorial therapies would ideally tackle different immune populations within the tumor bed and unleash immune cells’ anti-tumoral capacity to fight and eliminate MM, producing only mild toxicity. Hence, research work needs to continue aiming to find the perfect therapeutic combination. 

Despite all the challenges and limitations that immunotherapies pose, a wider impact on MM definitely requires a reinforcement of the immune anti-tumor mechanisms. 

## Figures and Tables

**Table 1:** Immune alterations found in the MM BM niche.

Immune Populations	Immune Dysfunction in the BM Microenvironment
Myeloid cells 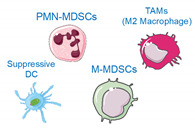	- Upregulation of inhibitory molecules not only by tumor cells, but also by macrophages and antigen-presenting cells, including PD-L1 [4] - Presence of tumor-associated macrophages (TAMs) and myeloid-derived suppressor cells (MDSCs), usually associated with poor prognosis [5,6] - Dendritic cells (DCs) are functionally defective due to the reduced expression of tumor antigens or HLA co-stimulatory molecules [7]
Lymphocytes 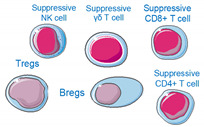	- Upregulation of inhibitory ligands, including PD-1 or TIGIT [8,9] - Infiltration of regulatory T and B cells (Tregs and Bregs, respectively), inhibiting T cells responses and promoting tumorigenesis [10,11] - Impaired induction of lymphocytes responses, due to a decrease in lymphocytes’ number or abnormal Th1/Th2 cytokine profile [12,13,14] - Decrease of the B cells compartment with altered cell differentiation and antibody response [15]

**Table 2:** Immune cells targeted by approved, breakthrough and future anti-myeloma immunotherapies.

Clinical Development Status	Immunotherapeutic Approach	Microenvironment Immune Cells Target	References
Approved immune strategies	ASCT	- Hematopoietic stem cells from own MM patients	Al Hamed et al., 2019 [16]
	AlloSCT	- Hematopoietic stem cells from healthy donors	Bashir et al., 2017 [22]
	IMIDs	- Stimulation of NK/NK T cells (ADCC) and activation of CD4+ and CD8+ T cells; - Suppression of Tregs	Quach et al., 2010 [30]
	PIs	- Inhibits osteoclasts and stimulates osteoblasts	Mothy et al., 2014 [157]
	mAbs	- Activation of both macrophages (ADCP) and NK cells (ADCC); - Suppression of Tregs, Bregs and MDSCs inhibitory activity	Kumar et al., 2016 [158]
Breakthrough immune therapies	CAR-T cells	- Genetically engineered T cells infused into the patient	June et al., 2018 [159]
	CAR-NK cells	- Genetically engineered NK cells infused into the patient	Rezvani, 2019 [95]
	BiTES/BiKES	- Genetically engineered T or NK cells infused into the patient, respectively	Caraccio et al., 2020 [99]
	ADCs	- Activation of both macrophages (ADCP) and NK cells (ADCC);	Bruins et al., 2020 [160]
	ICIs	- Breaks removal and activation of CD4+, CD8+, γδ T cells, NK cells, pDCs, and granulocytes in general; - Blocking MDSCs and Tregs inhibitory activity	Wang et al., 2020 [161] Kawano et al., 2017 [162]
Future approaches	MILs	- BM lymphocytes are reinfused	Borrello et al., 2016 [137]
	Vaccination strategies	- Previously educated DCs will present tumor antigens to T cells	Rhee, 2007 [163]
	Transgenic TCR	- Genetically engineered T cell infused into the patient, respectively	Schumacher, 2002 [164]
